# Impact of Catheter Management on Clinical Outcome in Adult Cancer Patients With Gram-Negative Bacteremia

**DOI:** 10.1093/ofid/ofz357

**Published:** 2019-09-28

**Authors:** Johny Fares, Melissa Khalil, Anne-Marie Chaftari, Ray Hachem, Ying Jiang, Hagop M Kantarjian, Issam I Raad

**Affiliations:** 1 Department of Infectious Diseases, Infection Control, and Employee Health, The University of Texas MD Anderson Cancer Center, Houston; 2 Department of Leukemia, The University of Texas MD Anderson Cancer Center, Houston

**Keywords:** bloodstream infection, cancer patients, central venous catheter, Gram negative

## Abstract

**Objective:**

Gram-negative organisms have become a major etiology of bloodstream infections. We evaluated the effect of central venous catheter management on cancer patients with gram-negative bloodstream infections.

**Method:**

We retrospectively identified patients older than 14 years with central venous catheters who were diagnosed with gram-negative bloodstream infections to determine the effect of catheter management on outcome. Patients were divided into 3 groups: Group 1 included patients with central line-associated bloodstream infections (CLABSI) without mucosal barrier injury and those whose infection met the criteria for catheter-related bloodstream infection; group 2 included patients with CLABSI with mucosal barrier injury who did not meet the criteria for catheter-related bloodstream infection; and group 3 included patients with non-CLABSI.

**Results:**

The study included 300 patients, with 100 patients in each group. Only in group 1 was central venous catheter removal within 2 days of bloodstream infection significantly associated with a higher rate of microbiologic resolution at 4 days compared to delayed central venous catheter removal (3–5 days) or retention (98% vs 82%, *P* = .006) and a lower overall mortality rate at 3-month follow-up (3% vs 19%, *P* = .01). Both associations persisted in multivariate analyses (*P* = .018 and *P* = .016, respectively).

**Conclusions:**

Central venous catheter removal within 2 days of the onset of gram-negative bloodstream infections significantly improved the infectious outcome and overall mortality of adult cancer patients with catheter-related bloodstream infections and CLABSI without mucosal barrier injury.

## Teaser

We evaluated cancer patients with different types of gram-negative bloodstream infection (BSI). Central venous catheter removal within 48 hours of the onset of BSI significantly improved the outcome in patients with definite catheter-related BSI and central line-associated BSI with no mucosal barrier injury.

## INTRODUCTION

Over the last 20 years, gram-negative (GN) organisms have become a major cause of bloodstream infections (BSIs), including central line-associated and catheter-related BSIs (CLABSIs and CRBSIs) and non-CLABSIs. This predominance mainly is seen in high-risk patients, such as cancer patients and intensive care unit patients [1–3]. Many studies have evaluated the common GN organisms seen in BSIs and their antimicrobial susceptibility patterns. *Escherichia coli* was found to be the predominant pathogen, with a high prevalence of multidrug resistance [1–6]. The Infectious Diseases Society of America (IDSA) recommends central venous catheter (CVC) removal in patients with long-term CVCs and uncomplicated GN CLABSIs; antimicrobial lock therapies are recommended if no other vascular access is available [7].

The US Centers for Disease Control and Prevention (CDC) introduced the concept of mucosal barrier injury (MBI) to recognize the potential gastrointestinal source of some BSIs, especially in cancer patients [8]. BSIs can be divided into 3 subgroups: CLABSI without MBI, which may represent true CRBSI; CLABSI-MBI, which are less likely to represent CRBSI; and non-CLABSIs in patients who do not have a CVC or in patients with another obvious infectious source [8].

The management of CVC in the setting of BSI remains challenging. Few studies have evaluated the effect of CVC management on the infectious outcome in cancer patients with GN BSIs [9, 10]. The aim of this retrospective study was to assess any differences in outcome on the basis of CVC management in cancer patients with CVCs and GN BSIs.

## METHODS

We searched the infectious control surveillance database and the microbiology laboratory database at The University of Texas MD Anderson Cancer Center (Houston, TX) from May 1, 2017, to May 31, 2018. We identified all patients with CVCs who had a documented BSI with a GN organism and were treated for it. We excluded patients with no CVC and those who were under the age of 14 years. We extracted patients’ data from the institution’s medical records, including their demographic characteristics, underlying malignancy, date of positive blood culture, presence or absence of MBI, type of GN organism isolated from culture, CVC characteristics, BSI management approach (systemic therapy and CVC management, such as CVC removal, exchange, or retention), and BSI outcome.

Patients were classified as having CLABSI or non-CLABSI according to the CDC criteria. Patients who had at least 1 positive blood culture with a GN organism and no apparent source of the BSI, other than the catheter, were classified as having a CLABSI. Those with a positive blood culture and an apparent BSI source other than the catheter were classified as having non-CLABSI [11]. We classified patients in the CLABSI group into 3 categories: CRBSI, CLABSI non-MBI, and CLABSI-MBI.

Mucosal barrier injury was defined as the presence of 1 of 3 conditions: (1) an absolute neutrophil count of <500 cells/mm^3^ on 2 separate days, within 3 days of bacteremia diagnosis; (2) a hematopoietic stem cell transplantation within 1 year of the positive blood culture with grade III or IV gastrointestinal graft-versus-host disease; and (3) severe diarrhea of ≥1 L within 24 hours of the positive blood culture or within the previous 7 days.

Patients with CLABSI also were evaluated to determine whether the infection met the IDSA criteria for CRBSI, whereby the BSI had to meet 1 of the following 3 criteria: (1) a 3-fold greater number of colonies of the organism from the CVC, compared to the same organism from the peripheral blood culture, drawn simultaneously; (2) differential time to positivity of at least 2 hours from the catheter-drawn blood culture to the peripheral blood culture; or (3) growth of the same organism from the percutaneous blood culture and the catheter tip [7].

We analyzed 3 groups of patients: group 1 (G1) included patients with CLABSI non-MBI and those whose infection met the CRBSI criteria. These patients were considered to have a definite or probable CLABSI. Group 2 (G2) consisted of patients with CLABSI-MBI whose infection did not meet the CRBSI definition; these patients were considered to have a possible CLABSI. Group 3 (G3) consisted of patients with non-CLABSI who had a CVC.

Our study included 300 patients, with 100 in each subgroup. Starting from May 1, 2018, we went backwards until we included 100 consecutive patients in each subgroup. We assessed catheter management (catheter removed or exchanged versus retained) at 2 and 5 days after the onset of bacteremia. We then determined the clinical and microbiologic outcome using the following measures: (1) defervescense at 3 days after the onset of bacteremia, (2) microbiologic resolution at 4 days after the onset of bacteremia, (3) occurrence of infection-related complications, (4) recurrence of BSI with the same organism, and (5) overall mortality, all within a follow-up period of 3 months after the onset of bacteremia. Infection-related complications included any deep-seated infections (such as endocarditis, osteomyelitis, or septic thrombophlebitis) or septic shock that was attributed to the bacteremia, from the day of the BSI onset until 3 months later. Recurrence of the BSI with the same organism within 3 months only was assessed in patients who had initially experienced microbiologic resolution. Associations between CVC management and outcomes were assessed separately in all 3 groups by univariate and multivariate analyses.

We obtained approval to conduct this retrospective study from our institutional review board and obtained a waiver of informed consent.

### Statistical Analysis

Categorical variables were compared using χ ^2^ or Fisher exact test, as appropriate. Continuous variables were compared using the Kruskal*-*Wallis test (for 3-group comparisons) and Wilcoxon rank sum test (for 2-group comparisons). If a significant result (*P* < .05) was detected for a test that compared 3 groups, then pairwise comparisons were performed, with α levels adjusted using Holm’s sequential *Bonferroni* adjustment to control for type I error [12]. A multivariate logistic regression model was used to evaluate the independent effect of CVC management on patient outcomes when the univariate analysis showed a significant (*P* < .05) or potentially significant association (*P* < .10). All tests were 2-sided, with a significance level of 0.05, except pairwise comparisons with an α adjustment. The statistical analyses were performed using SAS software version 9.3 (SAS Institute, Cary, NC).

## RESULTS

The demographic and clinical characteristics of the patients in the 3 groups are listed in Table 1. The age and sex distributions of the patients were similar. Most patients in G1 and G2 had hematologic malignancies as their underlying disease (66% and 90%, respectively), whereas in G3, most had a solid tumor (55%). Stem cell transplantation within 1 year of bacteremia and neutropenia were more common in G2 than in the other 2 groups (Table 1).

**Table 1.** Baseline Characteristics of Patients in All 3 Patient Groups

Characteristics	G1	G2	G3	*P* value	Pairwise Comparisons With Significant Differences
	(n = 100)	(n = 100)	(n = 100)		
Age (years), median (range)	56 (15–85)	57 (16–86)	61 (20–86)	.039	
Sex, male	65 (65)	61 (61)	51 (51)	.12	
Underlying disease				<.0001	G1 vs G2: *P* < .0001; G1 vs G3: *P* = .003; G2 vs G3: *P* < .0001
Hematologic malignancy	66 (66)	90 (90)	45 (45)		
Solid tumor	34 (34)	10 (10)	55 (55)		
Stem cell transplantation^a^	24 (24)	36 (36)	10 (10)	<.0001	G1 vs G3: *P* = .008; G2 vs G3: *P* < .0001
GVHD	13 (13)	3 (3)	9 (9)	.036	G1 vs G2: *P* = .009
Neutropenia	33 (33)	100 (100)	43 (43)	<.0001	G1 vs G2: *P* < .0001; G2 vs G3: *P* < .0001
ICU admission	2 (2)	2 (2)	11 (11)	.003	G1 vs G3: *P* = .01; G2 vs G3: *P* = .01
Polymicrobial infection^b^	20 (20)	15 (15)	12 (12)	.29	
Gram-negative organism					
*Escherichia coli*	18 (18)	66 (66)	44 (44)	<.0001	G1 vs G2: *P* < .0001; G1 vs G3: *P* < .0001 G2 vs G3: *P* = .002
*Enterobacter sp*	16 (16)	5 (5)	9 (9)	.03	G1 vs G2: *P* = .011;
*Pseudomonas aeruginosa*	20 (20)	0 (0)	14 (14)	<.0001	G1 vs G2: *P* < .0001; G2 vs G3: *P* = .0001
Other *Pseudomonas*	6 (6)	0 (0)	0 (0)	.004	
*Klebsiella* sp	14 (14)	18 (18)	23 (23)	.26	
*Stenotrophomonas maltophilia*	13 (13)	0 (0)	3 (3)	.0001	G1 vs G2: *P* = .0002; G1 vs G3: *P* = .009
*Acinetobacter* sp	9 (9)	0 (0)	0 (0)	<.0001	G1 vs G2: *P* = .003; G1 vs G3: *P* = .003
Other	22 (22)	12 (12)	13 (13)	.10	

Abbreviations: GI, gastrointestinal; GVHD, graft-versus-host disease; ICU, intensive care unit.

^a^Within 1 year prior to bacteremia.

^b^Ploymicrobial infections included infections with more than 1 gram-negative organisms and infections with both gram-negative and gram-positive organisms.

The common causative organisms significantly differed among the groups. *Escherichia coli* was significantly more common in G2, followed by G3, and then G1 (66%, 44%, and 18%, respectively; *P* < .0001). *Pseudomonas aeruginosa* and *Enterobacter* sp were more common in G1, followed by G3, and then G2. *Pseudomonas non-aeruginosa*, *S. maltophilia*, and *Acinetobacter* sp were more common in G1 than in G2 and G3, which had similar rates. *E. coli* and *Klebsiella* sp were the most common GN bacteria isolated in G2 and G3 (Table 1).

Most patients (73%) had a nontunneled catheter or a peripherally inserted central catheter (PICC) at the time of bacteremia. The rest (26%) had a tunneled catheter or a totally implantable port, and 2 patients had a hemodialysis catheter (data not shown). In terms of catheter management, 64% of patients in G1 had their CVC removed or exchanged within 2 days of bacteremia, 18% had their CVC removed or exchanged within 3–5 days, and 18% retained their CVC (Table 2). The rate of removal within 2 days was significantly higher in G1 than in G2 and G3 (45% and 25%, respectively; *P* < .0001) (Table 2). However, the rate of CVC removal increased after 5 days of bacteremia in all groups (G1, 82%; G2, 55%; and G3, 33%; respectively [G1 vs G2, *P* < .0001; G1 vs G3, *P* < .0001; G2 vs G3, *P* = .003]).

**Table 2.** Management and Outcome in All 3 Patient Groups

Characteristics	G1 (n = 100)	G2 (n = 100)	G3 (n = 93)^a^	*P* value	Pairwise Comparisons With Significant Differences
Catheter removal/exchange within 2 days^b^	64 (64)	45 (45)	23 (25)	<.0001	G1 vs G2: *P* = .007; G1 vs G3: *P* < .0001; G2 vs G3: *P* = .003
Catheter removal/exchange within 5 days^b^	82 (82)	55 (55)	31 (33)	<.0001	G1 vs G2: *P* < .0001; G1 vs G3: *P* < .0001; G2 vs G3: *P* = .003
Antibiotic treatment duration, median (IQR)	9 (6–13)	12 (8–16)	12 (8–15)	.001	G1 vs G2: *P* = .001; G1 vs G3: *P* = .003
Top 5 antibiotics used					
Amikacin	27 (27)	44 (44)	34 (37)	.04	G1 vs G2: *P* = .012
Cefepime	62 (62)	68 (68)	47 (51)	.04	G2 vs G3: *P* = .014
Ciprofloxacin	33 (33)	10 (10)	16 (17)	.0002	G1 vs G2: *P* < .0001; G1 vs G3: *P* = .012
Meropenem	30 (30)	59 (59)	49 (53)	<.0001	G1 vs G2: *P* < .0001; G1 vs G3: *P* = .001
Piperacillin-tazobactam	18 (18)	24 (24)	35 (38)	.007	G1 vs G3: *P* = .002
Receiving combination antibiotics^c^	53 (53)	62 (62)	61/93 (66)	.18	
Multidrug resistance	3 (3)	8/97 (8)	10/93 (11)	.10	
Defervescence^d^	70/82 (85)	71/89 (80)	54/73 (74)	.21	
Complications^e^	18 (18)	26 (26)	40/92 (43)	.0004	G1 vs G3: *P* = .0001; G2 vs G3: *P* = .011
Overall mortality^e^	9 (9)	16 (16)	27/92 (29)	.001	G1 vs G3: *P* = .0003
Microbiology resolution^f^	89/96 (93)	97/98 (99)	83/83 (100)	.006	G1 vs G3: *P* = .016
Recurrence^e^	5/95 (5)	7/96 (7)	6/83 (7)	.82	

Antibiotic treatment duration only reflects inpatient systemic therapy.

Abbreviation: IQR, interquartile range.

^a^Seven patients who died within 2 days of bloodstream infection were excluded from analysis, including 5 patients with catheters retained and 2 who died the same day when their catheters were removed.

^b^Since the date of positive blood culture.

^c^The top 5 antibiotics used in combination antibiotics were amikacin (33%), meropenem (31%), cefepime (25%), ciprofloxacin (13%), and piperacillin-tazobactam (12%).

^d^Within 3 days.

^e^Within 3 months of positive blood culture.

^f^Within 4 days.

The vast majority of patients in all 3 groups received systemic antibiotic therapy on the first day of bacteremia (the median time between positive blood culture and starting systemic therapy was 0 days in all 3 groups). The median time between starting antibiotic treatment and CVC removal or exchange was 1 day in all 3 groups (data not shown). Intravenous cefepime and meropenem were the most common antibiotics in all 3 groups (Table 2).

The univariate analyses showed that in G1 CVC removal within 2 days of BSI was significantly associated with higher rate of microbiologic resolution at 4 days compared to not doing so (delayed CVC removal [3 to 5 days] or CVC retention) (98% vs 82%, *P* = .006) and lower overall mortality rate at 3 months follow-up (3% vs 19%, *P* = .01) (Table 3). Furthermore, CVC removal within 2 days of BSI also was significantly associated with higher rate of microbiologic resolution (98% vs 72%, *P* = .002) and lower rate of mortality (3% vs 22%, *P* = .019) when compared to delayed CVC removal (3 to 5 days) alone. However, no significant association was seen between CVC removal within 2 days of bacteremia and defervescense at 3 days after the onset of bacteremia, occurrence of infection-related complications within 3 months, or recurrence of the BSI with the same organism within 3 months. Whereas for G2 and G3, CVC removal within 2 days of bacteremia was not significantly associated with any of the outcomes (data not shown). We then performed Cochran-Mantel-Haenszel tests to further evaluate the significant associations in G1 adjusting for different classes of empiric antibiotics use with different activity against bacteria embedded in biofilm (aminoglycoside vs fluoroquinolone) and found that the associations remained the same after the adjustment and the impacts of early CVC removal on outcomes were not affected by empiric antibiotics use (data not shown).

We conducted a multivariate analysis in G1 to rule out any possible confounding factors (Table 3). We considered all of the factors that might affect overall mortality and microbiologic resolution in our patient population, including age, underlying disease, ICU admission, graft-versus-host disease, neutropenic status, causative organism, polymicrobial infection, catheter type, start date, and duration of systemic antibiotic therapy and multidrug resistance. The association between CVC removal within 2 days of bacteremia and microbiologic resolution and overall mortality persisted (odds ratio [OR], 13.8; 95% CI, 1.6–120.0, *P* = .018 and OR, 0.13; 95% CI, 0.03–0.68, *P* = .016, respectively).

**Table 3.** Effect of Catheter Removal or Exchange on Outcomes in Patients With Catheter-Related or Center Line-Associated Bloodstream Infections Without Mucosal Barrier Injury (Univariate and Multivariate Analyses)

Outcome	Removal/Exchange Within 2 Days of pos. BCx, n (%)		Univariate Analysis	Multivariate Analysis
	Yes (n = 64)	No (n = 36)	Crude OR (95% CI), *P* value	Adjusted OR (95% CI), *P* value
Defervescence within 3 days of pos. BCx	51/58 (88)	19/24 (79)	1.92 (0.54–6.78), .32	^a^
Resolution within 4 days	62/63 (98)	27/33 (82)	13.8 (1.6–120.0), .018	13.8 (1.6–120.0), .018^b^
Recurrence within 3 months	3/62 (5)	2/33 (6)	0.79 (0.13–4.97), > .99	^a^
Complications within 3 months	10 (16)	8 (22)	0.65 (0.23–1.83), .41	^a^
Overall mortality within 3 months	2 (3)	7 (19)	0.13 (0.03–0.68), .016	0.13 (0.03–0.68), .016^b^

Abbreviations: 95% CI, 95% confidence interval; OR, odds ratio; Pos. BCx, positive blood culture.

^a^Multivariate logistic regression analysis was performed only for the outcomes with potential significant associations with catheter removal or exchange (*P* ≤ .10 on univariate analysis).

^b^Multivariate logistic regression analysis showed that catheter removal/exchange was the only factor that was independently associated with outcome. Because one-factor final model by multivariate analysis is equal to the model by univariate analysis containing the same factor, the odds ratio with 95% confidence interval and *P* value were the same between univariate and multivariate analysis.

## DISCUSSION

Our results indicate that CVC removal within 48 hours of the onset of bacteremia improved the infectious outcome of adult cancer patients with definite CRBSI and CLABSI non-MBI that was caused by GN organisms. However, CVC removal did not affect the outcome of patients with non-CLABSI or CLABSI-MBI. Moreover, in cases of GN bacteremia, CLABSI-MBI behaved similarly to non-CLABSI, but not CLABSI non-MBI or definite CRBSI, in terms of the impact of CVC management on outcome. CVC removal within 2 days of the onset of bacteremia in patients with CRBSI and CLABSI non-MBI (G1) was associated with a significantly better outcome (a lower overall mortality rate and a higher microbiologic resolution rate) than was delayed CVC removal (3–5 days after bacteremia) and CVC retention. This association persisted even after considering all of the confounding factors that might influence these 2 outcomes, including the start time and the duration of systemic antibiotic therapy.

Similar causative microorganisms also were found in the non-CLABSI and CLABSI-MBI groups. *E. coli* and *Klebsiella* sp were the most common GN bacteria isolated in both groups. Our data are consistent with those from previous studies that evaluated the most common causative GN organisms in similar populations [1, 9, 13]. These findings are explained by the fact that MBI increases the risk of translocation of the gut microorganisms, such as *E. coli* and *Klebsiella* sp, to the systemic circulation. *E. coli* was significantly more commonly isolated in patients with non-CLABSI and CLABSI-MBI than in those with CLABSI non-MBI or CRBSI. On the other hand, as expected, organisms that are not part of the normal gut flora, such as *Pseudomonas aeruginosa* and *non-aeruginosa*, *S. maltophilia*, and *Acinetobacter* sp, were significantly more commonly isolated in the CLABSI non-MBI and CRBSI group than in the other 2 groups. Causative microorganisms can help physicians and healthcare providers make decisions regarding line management in cases of GN bacteremia, especially in patients with MBI.

This study provides additional proof, in accordance with IDSA guidelines, that removing the CVC is the appropriate approach to managing CRBSIs and highly probable CLABSIs caused by GN organisms in cancer patients. It also supports the use of central line retention in patients with GN non-CLABSI [7].

A previous study by Hanna et al confirmed our finding by showing that early CVC removal improved the clinical outcome of cancer patients with GN CRBSI but did not affect the outcome of patients with GN non-CLABSI [9]. Similarly, another study conducted by Lee et al showed that delayed CVC removal (>3 days) in catheter-related GN bacteremia was associated with higher rates of overall mortality and persistent bacteremia. This association was not seen in patients with non-CLABSI [10]. Our results are in accordance with those of these previous studies. However, we found that CVC removal within 2 days was associated with a significant improvement in the clinical outcome compared to CVC removal after 2 days. Compared to the previous 2 studies, ours had a larger sample size. Moreover, patients with GN CLABSI-MBI whose infection did not meet the CRBSI criteria were studied as a separate group (G2). In other studies, these patients were combined with those who met the CRBSI definition [9], or they represented a very small percentage of the sample size [10]. CLABSI-MBI account for a large percentage of CLABSI cases in cancer patients [8, 13] and behaves differently than CRBSI and CLABSI non-MBI, as shown by our data. Thus, our study is a better representation of real-world experience and practice in a high-risk population, such as cancer patients, than are the previous 2 studies. In addition, to our knowledge, our study is the first to assess line management in cancer patients with GN CLABSI-MBI and the first to show that CVC removal does not improve the infectious outcome in these patients on the basis of real-world experience.

Our results show that in 45% of patients with CLABSI-MBI, the CVC was removed within 2 days of bacteremia; this percentage increased to 55% after 5 days. On the basis of our findings, we recommend against CVC removal in patients who have GN bacteremia that meets the criteria for CLABSI-MBI, but not CRBSI, and in whom the GN bacteria is a known gut organism. This subgroup of patients should be managed similarly to patients with non-CLABSI, and the CVC should be retained.

Unnecessary removal of CVCs in cancer patients is still a major drawback in the management of BSIs, knowing the financial burden and possible complications of CVC removal and insertion of a new CVC. A study conducted by Chaftari et al showed that CVCs were unnecessarily removed in 58% of cancer patients at MD Anderson with GN non-CLABSI between 2013 and 2014 [4]. Our study showed that 33% of cancer patients with GN non-CLABSI had their CVCs removed. This drop in the percentage of CVC removal in non-CLABSI patients is encouraging but is still high. Thus, it is crucial to educate physicians and health care providers in retaining the CVC in non-CLABSI cases as well as in CLABSI-MBI cases.

Our study is subject to some limitations. Given the retrospective design of the study, patients were not closely monitored and follow-up blood cultures were not obtained consistently. Furthermore, the patients were not treated according to a predefined protocol and may have received different antimicrobial agents or combination therapy. The type, combination, and duration of antimicrobial therapy, as well the CVC management, were not standardized and were left at the discretion of the treating physician. However, empiric antimicrobial therapy usually is adjusted according to the susceptibility profile of the organism.

In conclusion, CVC removal within 2 days of the onset of a GN BSI improves the infectious outcome in cancer patients with definite or highly probable line-related infections, such as GN CRBSI and CLABSI non-MBI. However, CVC removal does not affect this outcome in patients with GN CLABSI-MBI and non-CLABSI. Our results indicate that early CVC removal is not indicated in cases of non-CALBSI or when gut translocation secondary to MBI is highly suspected to be the source of bacteremia. Early CVC removal or the use of an effective antimicrobial lock therapy warrants further clinical trials in cancer patients with CVCs in the setting of GN CRBSI.
